# Effects of Orange Extracts on Longevity, Healthspan, and Stress Resistance in *Caenorhabditis elegans*

**DOI:** 10.3390/molecules25020351

**Published:** 2020-01-15

**Authors:** Jing Wang, Na Deng, Hong Wang, Tong Li, Ling Chen, Bisheng Zheng, Rui Hai Liu

**Affiliations:** 1 Overseas Expertise Introduction Center for Discipline Innovation of Food Nutrition and Human Health (111 Center), School of Food Science and Engineering, South China University of Technology, Guangzhou 510641, China; wj19950112@163.com (J.W.); daisynadeng@hotmail.com (N.D.); febzheng@scut.edu.cn (B.Z.); 2 Ministry of Education Engineering Research Centre of Starch & Protein Processing, Guangdong Province Key Laboratory for Green Processing of Natural Products and Product Safety, South China University of Technology, Guangzhou 510640, China; felchen@scut.edu.cn; 3 Department of Food Science, Stocking Hall, Cornell University, Ithaca, NY 14853, USA; tl24@cornell.edu; 4 Guangdong ERA Food & Life Health Research Institute, Guangzhou 510670, China

**Keywords:** orange extracts, *C. elegans*, anti-aging, antioxidant properties

## Abstract

Orange, with various bioactive phytochemicals, exerts various beneficial health effects, including anti-cancer, antioxidant, and anti-inflammatory properties. However, its anti-aging effects remain unclear. In this study, the *Caenorhabditis elegans* (*C. elegans*) model was used to evaluate the effects of orange extracts on lifespan and stress resistance. The results indicated that orange extracts dose-dependently increased the mean lifespan of *C. elegans* by 10.5%, 18.0%, and 26.2% at the concentrations of 100, 200, and 400 mg/mL, respectively. Meanwhile, orange extracts promoted the healthspan by improving motility, and decreasing the accumulation of age pigment and intracellular reactive oxygen species (ROS) levels without damaging fertility. The survival rates of orange extract-fed worms were obviously higher than those of untreated worms against thermal and ultraviolet-B (UV-B) stress. Moreover, the activities of superoxide dismutase (SOD) and catalase (CAT) were significantly enhanced while malondialdehyde (MDA) contents were diminished. Further investigation revealed that worms supplemented with orange extracts resulted in upregulated levels of genes, including *daf-16*, *sod-3*, *gst-4*, *sek-1*, and *skn-1*, and the downregulation of *age-1* expression. These findings revealed that orange extracts have potential anti-aging effects through extending the lifespan, enhancing stress resistance, and promoting the healthspan.

## 1. Introduction

The aging process is accompanied by a progressive decline of physiological integrity, eventually leading to increased sensitivity to stress and impaired function. The deterioration of aging is a major risk factor of the global burden of chronic diseases, like neurodegenerative diseases, cancer, diabetes, and cardiovascular disorders [1]. Previous research has indicated that increased consumption of fruit and vegetables can effectively reduce the risk of some functional declines associated with aging, which is associated with the additive and synergistic interactions among phytochemicals existing in whole fruit and vegetables [2]. Bioactive non-nutrient plant phytochemicals, especially phenolicsand flavonoids, have been linked to many biological properties, such as antioxidant, anticancer, and anti-inflammatory properties, which could be potential sources of anti-aging agents due to their excellent biological activities [3].

The nonparasitic nematode *C. elegans*, a simple and useful in vivo model, has been widely used for screening bioactive compounds for the anti-aging effect [4]. *C. elegans* has the advantages of a simple physiological structure, small body size, easily cultured, short life cycle, large number of offspring, and specific gene sequences [5]. The genes are highly conserved and distinct, with 60% to 80% of the genes in *C. elegans* being similar to human gene homologs [6]. The insulin/ insulin-like growth factor signaling (IIS) pathway and mitogen-activated protein kinase (MAPK) pathway are both important signal pathways in nematodes, which are associated with lifespan and stress resistance [7]. Meanwhile, the changes of behavior and physiological indicators of healthspan, such as the deterioration of muscle, resistance to environment stress, and degeneration of the nervous system, are in a similar manner to those of humans [8]. Thus, these features make *C. elegans* an ideal model to clarify the anti-aging effect of bioactive compounds on the healthspan and lifespan.

Some studies have suggested that ROS might induce oxidative stress, thus reducing the lifespan of *C. elegans* beyond a certain concentration range [9]. In addition, antioxidants that are able to scavenge overproduced radicals may extend the lifespan and delay the deterioration of aging [10]. A previous report showed that phytochemicals extracted from blueberry could promote stress tolerance and longevity via dauer formation (DAF)-16 in *C. elegans* [11]. *Anacardium occidentale* leaf extracts containing a number of polyphenolic antioxidants enhanced the survival rate of nematodes under oxidative stress via the DAF-16/FOXO (Forkhead box O) and SKN-1/Nrf-2 (Nuclear factor erythroid 2-related factor 2) signaling pathways [3]. 4-Hydroxybenzoic acid isolated from *Veronica peregrina* prolonged the lifespan of *C. elegans* via SIR-2.1/SIR2-mediated DAF-16/FOXO activation, independent of dietary restriction and the IIS pathway [12]. *Rosa rugosa* aqueous polyphenol extracts had anti-aging effects on *C. elegans*, which might be attributed to its powerful antioxidant effects [13].

Orange is a widely available and economically significant fruit containing a large number of polyphenolic secondary metabolites with potent antioxidant and antiproliferative activities [14]. Citrus flavanone naringenin was shown to prevent myocardial cells from senescence through the modulation of cell cycle regulators, ROS levels, and mitochondrial metabolic activities [15]. The juice of bergamot (*Citrus bergamia* Risso et Poiteau) counteracted the senescence processes in senescent H9c2 cells and old mice [16]. All findings revealed that orange phytochemicals might have anti-aging effect on lifespan. However, little is known about the effect of fresh orange flesh on the lifespan in vivo and the underlying mechanism.

Herein, *C. elegans* was used as an in vivo model to investigate whether orange extracts have beneficial effects on lifespan, healthspan, or stress resistance. Moreover, the underlying mechanism involved in the effect on lifespan with orange extracts was also determined.

## 2. Results

### 2.1. Chemical Characterization of Orange Extracts

**Figure 1:**
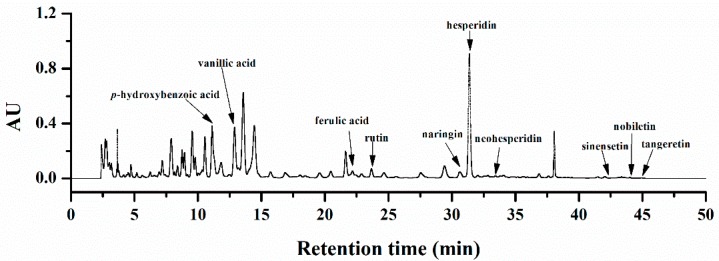
HPLC chromatogram of phytochemicals of orange extracts at 283 nm.

Eleven compounds, including seven flavonoids and four phenolic acids, were identified in the high-performance liquid chromatography (HPLC) profile of orange extracts (Figure 1). Hesperidin was the predominant phenolic compound (50.05 ± 10.18 μg/g FW), followed by vanillic acids, *p*-hydroxybenzoic acid, and rutin. Several characteristic citrus flavones, such as nobiletin, naringin, tangeretin, neohesperidin, and sinensetin, were also observed in the orange extracts.

### 2.2. Orange Extracts Prolonged the Lifespan of Wild-Type C. elegans

**Figure 2:**
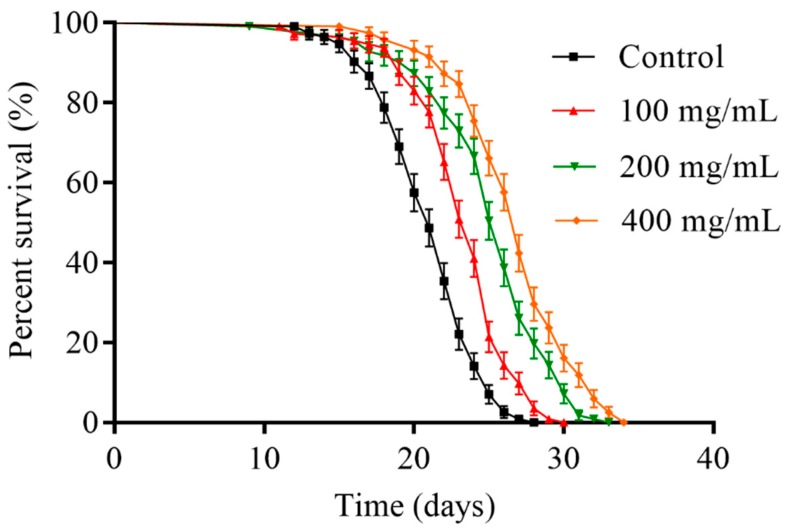
Effect of orange extracts on the lifespan of N2 wild-type *C. elegans*. Day 1 adult nematodes at the L4 stage (fourth larval stages) were treated with and without orange extracts (0, 100, 200, or 400 mg/mL) at 20 °C. The survival was counted starting from day 1 of adulthood to death. Supplementation with orange extracts significantly increased the survival rate of nematodes compared to those not treated with orange extracts (control). The experiment was performed in triplicate.

The N2 wild-type *C. elegans* cultured on different concentrations of orange extracts (100, 200, and 400 mg/mL) were monitored to investigate whether orange extracts influenced the lifespan of *C. elegans*. The survival curves and results are shown in Figure 2 and Table 1. The results illustrated that worms treated with different concentrations of the sample showed right-shifted survival curves, especially the 400 mg/mL orange extract treatment group. The control group of nematodes without orange extracts had a mean lifespan of 20.91 ± 1.60 days under the proper survival conditions. Compared with the control, the highest concentration of the 400 mg/mL group significantly extended the mean lifespan to 26.38 ± 2.06 days and the survival rate increased by 26.2%. Subsequently, the mean lifespan of the 200 mg/mL group was 24.67 ± 2.26 days and this group showed an 18.0% increase compared with the control. The 100 mg/mL group had a relatively lower increase, with a 10.5% and little longer mean lifespan of 23.10 ± 1.95 days. As illustrated above, orange extracts could markedly prolong the lifespan of *C. elegans* strain N2 in a dose-dependent manner.

### 2.3. Reproduction Capacity of C. elegans Treated with Orange Extracts

**Figure 3:**
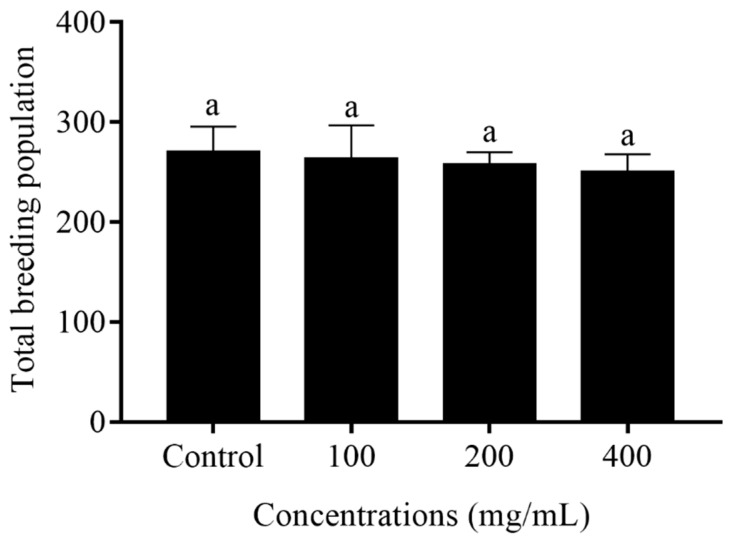
Effect of orange extracts on the total breeding population in N2 wild-type *C. elegans*. Nematodes were incubated in the presence of orange extracts at different concentrations of 0, 100, 200, or 400 mg/mL. The number of offspring of each worm were counted until the parental nematode stopped producing progeny (about day 6). All data were determined in triplicate (mean ± SD, *n* = 3). The bars with different letters indicate statistical significance.

Reproduction capacity, as an aging-related factor, was further used to check the influence of orange extracts on N2 wild-type *C. elegans*. Nematodes generally have a high reproductive capacity in the first 4 days of the L4 phase, and with the increase of life span, the reproductive capacity gradually decreases until the fifth day. Therefore, we determined the total offspring number within the first 5 days of the L4 phase to represent the reproductive capacity. As illustrated in Figure 3, there were no significant differences in the total breeding population among the worms fed with various concentrations of orange extracts and control worms, ranging from 250 ± 17 eggs/worm in the 400 mg/mL orange extract-treated group to 270 ± 25 eggs/worm in the control group.

### 2.4. Effects of Orange Extracts on Physiological Responses of C. elegans

**Figure 4:**
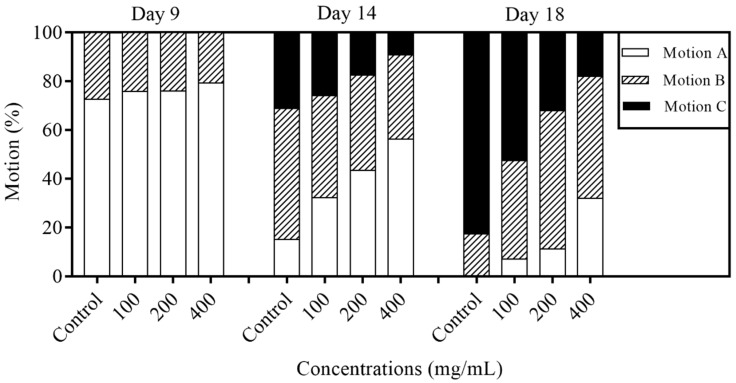
Effect of orange extracts on the motility of *C. elegans*. Day 9, day 14, and day 18 animals were assessed for their motion ability. Motility was classified into three groups: Motion A, nematodes moved freely; motion B, nematodes moved slowly only in response to stimulation; and motion C, nematodes only moved their heads or tails after prodding. Treatment with orange extract significantly delayed the decline in motility at all ages in a dose-dependent manner. The experiments were performed in three independent assays.

A series of physiological representations, including movement behavior, body length, and age pigment, were used as aging-associated indicators to assess the healthspan of *C. elegans*. The motility characteristics of nematodes treated with orange extracts were evaluated at early, moderate, and late life stages (on day 9, 14, and 18), respectively (Figure 4). Obviously, when worms were in an early stage of the life process (on the ninth day), over 70% of the animals were still assessed as motion A worms regardless of whether the worms were fed the with or without orange extracts. On day 14, the motion A animals decreased significantly whereas motion B and motion C worms increased dramatically when compared with the situation on day 9. Notably, the proportion of motion A ascended with the increasing concentrations of orange extract treatments (32%, 44%, and 56% in the 100, 200, and 400 mg/mL group, respectively). By day 18, the orange extract-fed worms still exhibited better movement capacity in a dose-dependent manner compared with the control group, such that 32% of the animals treated with 400 mg/mL of orange extracts still moved spontaneously (motion A) while 83% of the control were assessed as motion C. Overall, the orange extract treatment potently improved the movement behavior of *C. elegans* in a dose-dependent manner.

**Figure 5:**
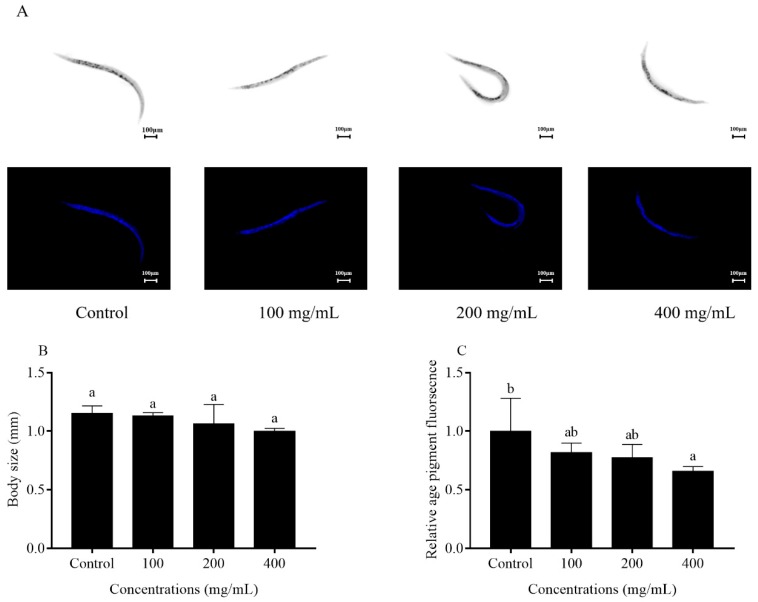
Effect of orange extracts on body size and age pigment accumulation in *C. elegans*. Wild-type N2 animals were treated without or with 100, 200, and 400 mg/mL orange extracts for 5 days at 20 °C. (**A**) Representative pictures of the body size and age pigment accumulation of N2 worms. Body size (**B**) and relative fluorescence of the age pigment (**C**) were quantitated by Image J software. Bars with different letters indicate statistical significance (*p* < 0.05, mean ± SD, *n* = 3).

Then, body size was assessed among the control and orange extract-treated groups. The results showed that there were no significant differences in the body size among the groups, indicating orange extract exposure did not potently affect the body size of the worms (Figure 5B). Age pigment gradually accumulated in the aged nematodes so that the fluorescence could reflect the aging level of the worms. We found that orange extract treatment could improve age pigment aggregation. Compared with the control group, age pigment fluorescence decreased by 18.3%, 22.5%, and 34.4% at 100, 200, and 400 mg/mL of orange extract treatment, respectively (Figure 5C).

### 2.5. Orange Extracts-Treated C. elegans Exhibited Enhanced Resistance of Heat Shock and UV-B Radiation

**Figure 6:**
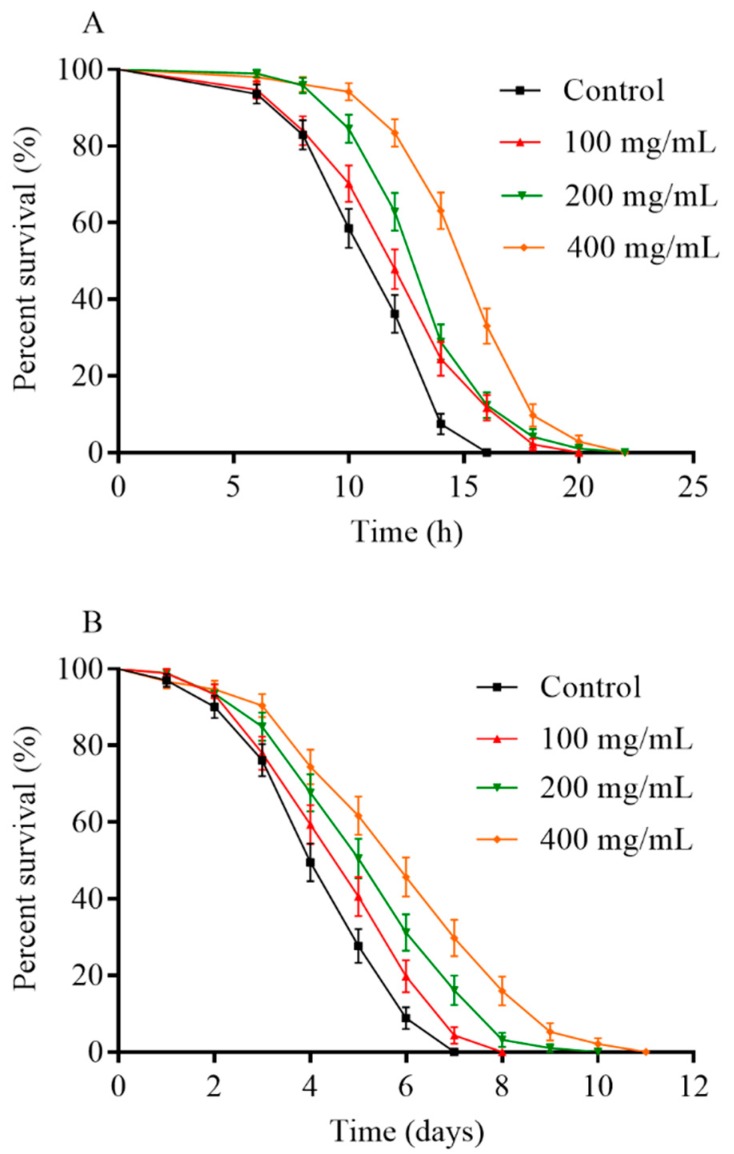
Effect of pretreatment with orange extracts on resistance to stress in N2 wild-type *C. elegans*. Nematodes were treated with various concentrations of orange extract (0, 100, 200, 400 mg/mL) as young adults for 5 days at 20 °C and then exposed to different stressors: (**A**) 35 °C thermal shock (N ≥ 95). (**B**) UV-B radiation at 120 mJ/cm^2^ (N ≥ 92). The experiment was performed in triplicate.

Lifespan extension is often associated with enhanced stress resistance in *C. elegans* [17]. We firstly measured the mean lifespan of *C. elegans* in thermal stress conditions at 35 °C to investigate the influence of orange extract-mediated heat tolerance (Figure 6A, Table 2). The mean lifespan of the control worms was 11.58 ± 1.02 h under the thermal stress conditions at 35 °C, and the treated groups lived an average of 12.71 ± 0.99 h (maximum of 19.33 ± 1.15 h), 13.81 ± 1.24 h (maximum of 21.33 ± 1.15 h), and 15.68 ± 1.09 h (maximum of 21.33 ± 1.15 h) following the orange extract treatment at different concentrations of 100, 200, and 400 mg/mL, respectively. In comparison with the untreated control group, the mean survival time increased by 9.8%, 19.3%, and 35.5% after treatment with 100, 200, and 400 mg/mL orange extracts. The results revealed that orange extract supplementation evidently mitigated heat stress and increased the survival rates of the worms in a dose-dependent manner. Subsequently, we determined whether orange extracts were able to enhance the UV-B radiation stress resistance in N2 *C. elegans* (Figure 6B, Table 3). The survival rates of animals pretreated with orange extracts had a similar trend to the heat shock assay. Orange extract-fed *C. elegans* survived significantly longer after UV radiation (120 mJ/cm^2^) than N2 control animals. The 400 mg/mL orange extracts had a much greater effect, with a 35.6% increase in the mean lifespan compared with thee control. The mean lifespans of worms after ultraviolet-B (UV-B) treatment were 4.53 ± 0.52 days (maximum of 6.67 ± 0.58 days), 4.94 ± 0.71 days (maximum of 7.67 ± 0.58 days), 5.52 ± 0.77 days (maximum of 9.00 ± 1.00 days), and 6.16 ± 0.90 days (maximum of 10.33 ± 0.58 days) for the 0, 100, 200, and 400 mg/mL orange extracts, respectively. All results indicated that orange extracts exhibited tolerance of UV-B radiation.

### 2.6. Orange Extracts Decreased Intracellular ROS Accumulation in C. elegans

**Figure 7:**
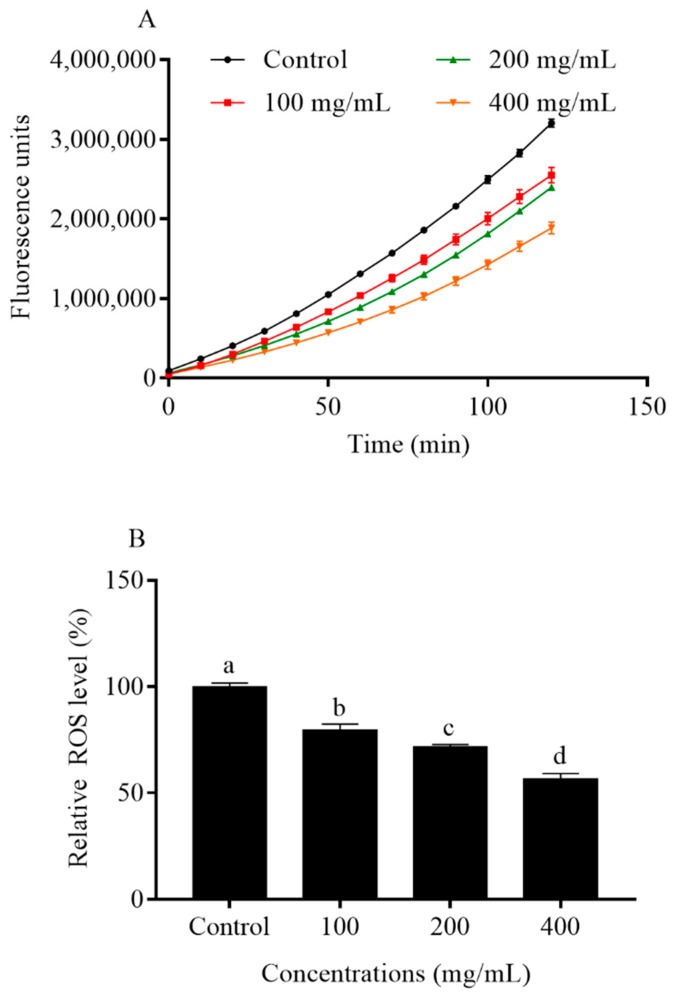
Effect of orange extracts on intracellular ROS accumulation in *C. elegans*: (**A**) Effect of orange extracts on ROS fluorescence in *C. elegans*. (**B**) Relative ROS levels compared with the control. Bars with different letters indicate statistical significance (*p* < 0.05, mean ± SD, *n* = 3).

Oxidative stress induced by ROS accumulation is a major factor limiting the lifespan of *C. elegans* [12]. Reducing the overproduction of intracellular ROS exerts a positive impact on oxidative stress resistance. In our study, the levels of ROS in N2 *C. elegans* were determined by the ROS indicator Dichlorofluorescin-diacetate (DCFH-DA). DCFH-DA, as a membrane-permeable molecule without fluorescence, is oxidized into fluorescent 2′,7′-dichlorofluorescein (DCF) in the presence of intracellular ROS, thus the levels of intracellular ROS can be determined by monitoring the variation of the fluorescent intensity of DCF. As Figure 7 illustrated, 5-day pre-treatment with orange extract significantly reduced ROS at all concentrations. The ROS levels were respectively reduced by 20.5%, 28.4%, and 43.3% among orange extract-treated groups at 100, 200, and 400 mg/mL compared with the control nematodes. The results indicated that supplementation with orange extracts inhibited intracellular ROS formation in wild-type nematodes in a dose-dependent manner.

### 2.7. Antioxidant Enzyme Activities and MDA Contents in C. elegans

**Figure 8:**
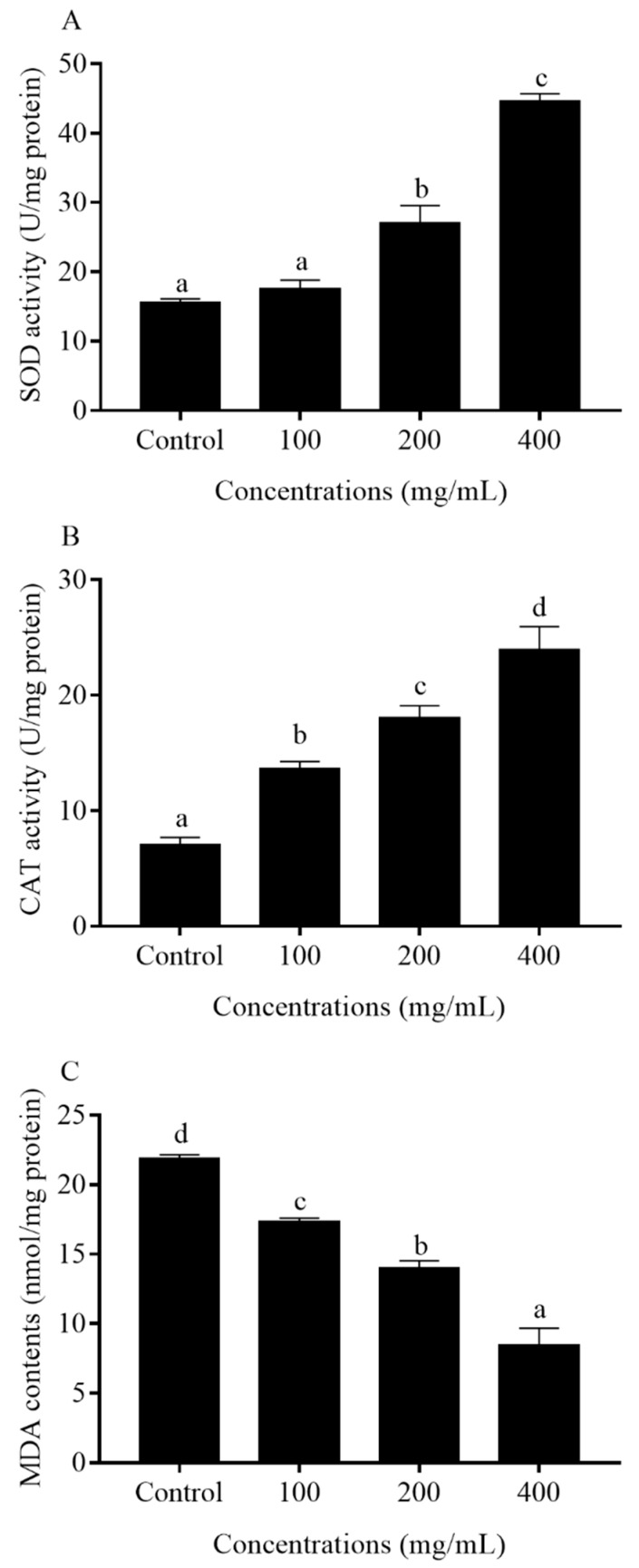
Effect of orange extracts on antioxidant enzymes and MDA contents in *C. elegans* (mean ± SD, *n* = 3): (**A**) SOD activity; (**B**) CAT activity; and (**C**) MDA content. Bars with different letters indicate statistical significance (*p* < 0.05, mean ± SD, *n* = 3).

The effect of orange extracts on the activities of SOD and CAT and the contents of MDA were analyzed from orange extract-treated *C. elegans* (Figure 8). After 5-day exposure to orange extracts, the 200 and 400 mg/mL orange extract-treated groups led to a 1.7- and 2.9-fold increment in SOD activity compared with the control worms. The 100 mg/mL group slightly increased SOD activities (1.1-fold) compared with the control. Compared with the control, the CAT activities of the 100, 200, and 400 mg/mL orange extract-treated groups also increased by 0.9-fold (100 mg/mL, *p* < 0.05), 1.4-fold (200 mg/mL, *p* < 0.05), and 2.2-fold (400 mg/mL, *p* < 0.05), respectively. Therefore, the abilities of orange extracts to scavenge for O_2_^−^ and H_2_O*_2_* could be found in *C. elegans*. The generation of MDA is a mark of oxidative damage of an organism. Since orange extracts were able to promote oxidative stress resistance in nematodes, we determined the MDA levels in the N2 *C. elegans* strain. The MDA contents were 20.7% to 61.1% lower in the orange extract-treated groups than those in the control group. The findings indicated that orange extracts played a crucial role in reducing the end product of lipid peroxidation (MDA) of aged worms.

### 2.8. Orange Extract Treatment Regulated the Expression of Messenger RNA (mRNA) in C. elegans

**Figure 9:**
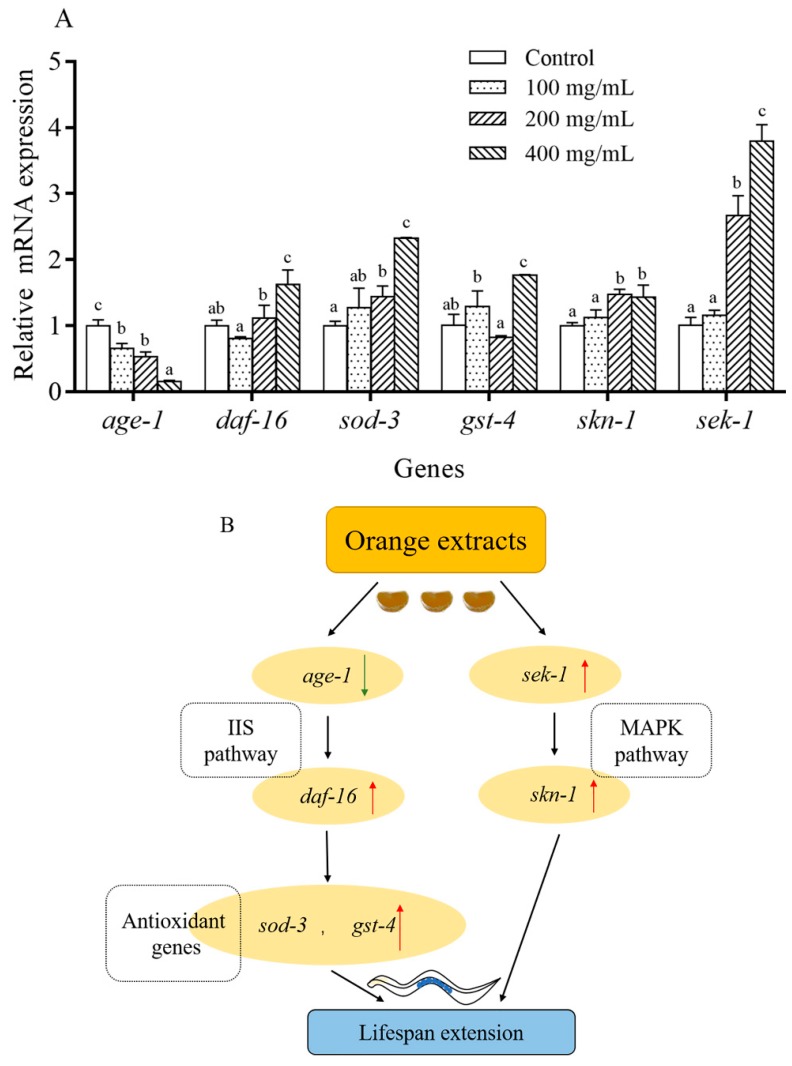
Effect of orange extracts on age-related gene expressions in *C. elegans*. (**A**) The mRNA expressions of age-related genes. Bars of the same gene with different letters indicate statistical significance (*p* < 0.05, mean ± SD, *n* = 3). (**B**) Possible molecular mechanism of the anti-ageing effect of orange extracts.

The results above indicated that orange extract effectively mediated the extension of the worms’ lifespan. To unravel the mechanism of this effect at the transcription level, we measured the relative expression levels of stress resistance and longevity-related genes, including *age-1* (Phosphatidylinositol 3-kinase age-1), *daf-16* (Fork-head domain-containing protein), *sod-3* (Superoxide dismutase [Mn] 2), *gst-4* (Glutathione S-transferase 4), *sek-1* (Dual specificity mitogen-activated protein kinase kinase sek-1), and *skn-1* (BZIP domain-containing protein), through quantitative Real-time PCR (RT-PCR) (Figure 9A). The expression levels of some key genes (*age-1*, *daf-16*, *sod-3*, and gst-4) in the IIS pathway, which is an evolutionarily conserved pathway involved in regulating the lifespan, stress resistance, and ROS formation in *C. elegans,* were determined. The orange extract treatment upregulated the relative expression levels of *daf-16* by 0.81 to 1.63-fold but downregulated *age-1* by 0.65-fold to 0.16-fold at different concentrations compared with the control. The expression levels of the downstream target genes *sod-3* and *gst-4* ranged from 1.28- to 2.32-fold and from 1.30- to 1.77-fold at different doses of orange extract, respectively. The mRNA expression of *sek-1* and *skn-1* in the MAPK pathway, which plays a crucial role in stress resistance and lifespan regulation in *C. elegans*, was also analyzed in our study. We found that orange extracts dramatically elevated the expression of *skn-1*, ranging from 1.13- to 1.44-fold, and *sek-1*, ranging from 1.16- to 3.79-fold. These results showed that orange extract treatment increased the expression levels of *daf-16*, *sod-3*, *gst-4*, *sek-1*, and *skn-1* and suppressed *age-1* expression. 

## 3. Discussion

Orange is one of the most consumed fruits all over the world with rich phytochemicals [18]. Previous studies illustrated that the phytochemicals extracted from orange have various biologically active properties, including antiproliferative, antioxidant, and anti-inflammatory activities [19,20,21]. Orange products and phytochemicals derived from whole orange have been shown to extend the mean lifespan of some in vivo models, such as *Saccharomyces cerevisiae* [22], *Drosophila melanogaster* [23], and rodents [24]. For instance, Sun et al. found that hesperidin (10 μM) extracted from the *Citrus* genus increased the lifespan of K6001 yeast by 36.9%, which could be attributed to inhibition of reactive oxygen species and regulation of the *sir2* and *uth1* genes [22]. Zahira and coworkers showed that proper concentrations of orange juice and its predominant components, including hesperidin and limonene, increased the healthspan of living flies [23]. In Chikakos’ research, lemon polyphenols prolonged the lifespan of senescence-accelerated mouse prone 1 (SAMP1) by approximately 3 weeks and delayed the increment of locomotor atrophy and aging-related scores [24].

In this study, the *C. elegans* model was chosen to investigate the effect of orange extract treatment on the worm’s lifespan, because of its selective advantage in aging research, drug toxicity testing, and abiotic stress studies [25]. We showed for the first time that orange extract extended the lifespan of *C. elegans* in a dose-dependent manner. In addition, in the preliminary experiments, we determined that the concentrations of all the orange extracts used in the present study did not inhibit the growth of *Escherichia coli OP50* (*E. coli* OP50). Therefore, it is unlikely that orange extracts prolonged the lifespan of *C. elegans* through its antimicrobial effect. The orange extract treatment obviously increased the mean lifespan of N2 *C. elegans* by 26.2% at the concentration of 400 mg/mL, which was consistent with the findings of Ricardo et al. [26]. It was suggested that 2% pasteurized “Cara Cara” orange juice increased the mean lifespan of *C. elegans* by up to 29.5% compared to the untreated group [26]. Some investigators claim that the synergistic interactions among various compounds make the whole food elicit the maximum anti-aging effect [11]. In our previous study, Vayndorf et al. found that whole apple extract extended *C. elegans* mean lifespan by 39%, which was greater than its major compounds, like quercetin (up to a 15% increase in the mean lifespan of *C. elegans*) and Epigallocatechin gallate (EGCG) (up to a 10% increase in the mean lifespan of *C. elegans*) [27]. Therefore, the anti-aging effects found in orange extracts might be associated with the interaction of a range of various phytochemicals. At the same time, the relevant antioxidant enzyme activities (SOD and CAT), levels of intracellular ROS, and contents of MDA changed accompanied by an increase in the life expectancy of *C. elegans*. The data available showed that the activities of SOD and CAT were significantly increased after treatment with orange extract while the contents of MDA and levels of intracellular ROS were reduced. Since phenolic compounds acting as good antioxidants able to scavenge free radicals and upregulate antioxidant enzyme activities are abundant in orange extracts, we inferred that these phenolics absorbed by nematodes resulted in the enhancement of the antioxidant properties in vivo and the mitigation of cellular damage caused by ROS, thus lengthening the lifespan of *C. elegans*. The anti-aging effects influenced by orange extracts significantly broadened our knowledge of the biological effects of orange phytochemicals.

Some researchers have demonstrated that reductions in reproduction could prolong the life process in *C. elegans* [12]. In this study, the results showed that no statistically significant differences were found between the control and orange extract treatment on fecundity, which meant orange extracts did not change the reproductive capacity while extending the lifespan of *C. elegans*. A series of physiological functions, including the locomotor capacity, age pigment, and body length, have a connection with the lifespan [28,29]. We observed that worms supplemented with orange extracts showed more youthful and energetic mobility than control worms at different stages of the life process. At the late stage on day 18, there were still some motion A animals in the different orange extract concentration treatment groups, whereas the motility of control worms was only scored as motion B or C. The age pigment, an aging-related marker, was used to reflect the degree of aging. The accumulation of age pigment in *C. elegans* was reduced dose dependently by orange extracts, and the most significant effect in reducing the accumulation of age pigment was shown at 400 mg/mL (decreased by 34.4% compared with the control). Since many reports claimed that the pre-treatment of antioxidants could decrease age pigment accumulation, [3] we inferred that orange extract downregulation of age pigment levels might be associated with its strong antioxidant potential in *C. elegans* (Figure 5 and Figure 8). In addition, there was no obvious effect in the body length of orange extract-treated and untreated worms. All the results suggested that orange extracts had a beneficial effect on not only the lifespan but the healthspan of worms.

The genetic make-up is dominant in determining the lifespan of organisms while the influence of environmental factors, such as multiple stressors and dietary supplementation, on lifespan cannot be ignored [30,31]. Subsequently, we exposed the different pretreated worms to heat shock or UV-B radiation conditions. The *C. elegans* survival rates were greatly improved following the thermal stresses at 35 °C and 120 mJ/cm^2^ of UV-B radiation by orange extract treatment. Orange extracts enhanced the thermotolerance and resistance to UV-B radiation, which meant that orange extracts might improve the ability of *C. elegans* to deal with poor living environments. Zhang et al. [13] found that an aqueous polyphenol extract from *Rosa rugosa* tea influenced both the thermal stress resistance and mean lifespan of worms, which was similar to the result that the increase of the mean lifespan of *C. elegans* was accompanied by an improvement in thermotolerance mediated by orange extracts. UV-B radiation leads to DNA damage [32], which could be repaired by the bioactive compounds found in citrus fruits, like naringenin [33]. Thus, the benefit of the enhancement of UV-B stress resistance by orange extracts might be associated with the phytochemical profiles of orange extracts. In the present study, orange extracts significantly increased the lifespan of *C. elegans* under normal and stress conditions.

The results of the transcriptional analysis illustrated that orange extracts activated the expressions of *age-1* and *daf-16* genes in the IIS pathway, *sek-1* and *skn-1 genes* in the MAPK pathway, and antioxidant genes, including *sod-3* and *gst-4* (Figure 9B). The IIS pathway is a highly conservative and elaborate lifetime regulator, which plays a key role in development, metabolism, and aging in *C. elegans* [3]. This signaling pathway is activated by the translocation of DAF-16 to the nucleus [34]. The upregulation of *daf-16* was combined with the downregulation of *age-1* and activation of downstream target antioxidant genes *sod-3* and *gst-4* to pave the way for post-transcriptional exploration in the IIS pathway in the future. Moreover, the MAPK pathway is an integral part of mitochondrial detoxification and lifespan regulation [35]. The expression of *skn-1* and *sek-1* were upregulated in this pathway, especially *sek-1*, after treatment with orange extracts. *Sek-1* is known to be required for translocating cytoplasmic SKN-1 to intestinal nuclei under oxidative stress conditions and *skn-1* regulates downstream stress-responsive gene expression [7]. A previous study found that *skn-1* mutant *C. elegans* showed decreased resistance to oxidative stress and a shortened lifespan, which indicates that SKN-1 also modulates lifespan extension in addition to stress resistance [36]. The activation of both genes also provides a basis for research of the MAPK pathway.

## 4. Materials and Methods

### 4.1. Materials and Reagents

Dichlorofluorescin-diacetate (DCFH-DA) and 5-fluoro-2′-deoxyuridine (FUDR) were purchased from Sigma-Aldrich Inc. (St. Louis, MO, USA). Wild-type N2 *C. elegans* were purchased from the Caenorhabditis Genetics Center (CGC), (University of Minnesota, Minneapolis, MN, USA). *Escherichia coli OP50* (*E. coli* OP 50) was obtained from the Wu laboratory, Huazhong University of Science and Technology (Wuhan, China). The assay kits of malondialdehyde (MDA), catalase (CAT), superoxide dismutase (SOD), and the total protein were purchased from Nanjing Jiancheng Technology Co., Ltd. (Nanjing, China). All the other chemicals and solvents were of analytical grade.

### 4.2. Sample Preparation

Fresh oranges with the same degree of ripening were purchased from a local supermarket in September 2018. The phytochemicals were extracted from fresh orange with 80% acetone according to a previous report [37]. The mixture was centrifuged to collect all the supernatant. Subsequently, the supernatant was evaporated at 45 °C and then reconstituted with deionized water. Finally, the major phytochemicals of the orange extracts were identified through a Waters series HPLC system [19]. Orange extracts were stocked at a concentration of 1 g/mL based on the fresh weight of orange and frozen at −80 °C for further analysis.

### 4.3. C. elegans Cultivation

Wild-type N2 *C. elegans* were cultivated on nematode growth medium (NGM) with living *E.coli* OP 50 as a food resource and incubated at 20 °C by referring to the methods in our laboratory [11]. A bleaching buffer containing 5% NaClO and 5 M NaOH was used to lyse gravid adult worms to release eggs. Then, eggs were collected with M9 buffer to obtain age-synchronized nematodes [27].

### 4.4. The Lifespan Assay of C. elegans

Growing synchronized nematodes at the L4 stage were utilized for a lifespan assay on NGM plates (at least 30 nematodes per plate, and a total of 3 plates per control and treatment) covered with *E.coli* OP50 [38]. *C. elegans* were divided into experimental groups treated with various concentrations of orange extracts (100, 200, and 400 mg/mL) and a control group with no extracts on the NGM plates. The orange extracts were directly added on top of the solid plate. In total, 50 µM 5-fluoro-2′-deoxyuridine (FUDR) added to the plate was used to prevent the reproduction of the progeny. The day when worms were first treated with orange extracts was considered as day 1 of the nematode lifespan. Worms were transferred to new treatment plates every other day and the numbers of dead and surviving nematodes were scored daily until all nematodes were dead. Dead worms that failed to respond to gentle touch with platinum wire were removed from the agar plates. Individuals producing worm bags, becoming desiccated, crawling on the side of the plate, or exhibiting abnormal death were excluded from the experiment in time. The survival curve plotted as a percentage of the survival and statistical analyses were carried out using GraphPad software. Data were reported as mean ± SD. The experiments were independently repeated at least three times.

### 4.5. Motility Assay

Worms were treated with or without orange extracts as in the lifespan assay described above. At least 30 nematodes per plate were counted using a stereo microscope on the 9th, 14th, and 18th day of adulthood. The locomotion phenotypes of worms were judged by a mechanical stimulus with a platinum wire, and were determined to classify the motility grades. Motion A worms were classified as those that moved smoothly without touch. Worms that were unable to move independently but were moving heads or tails after stimulus were considered as motion C. Motion B worms were between A and B [39].

### 4.6. Reproduction Assay

Gravid nematodes at the L4 stage were allotted to NGM plates without FUDR to examine the reproduction capacity [28]. Only one nematode was located on each plate. In addition, the day when the worms were treated with or without orange extract was regarded as day 1 of the reproduction assay. From day 1, this parental worm was transferred daily to a new fresh plate without FUDR until the worm stopped producing offspring (on day 6). The number of offspring produced by each worm across the breeding period were counted and summed to obtain the total number of offspring. Besides, the average number of offspring for each concentration was analyzed. Each experiment was carried out for a total of three times.

### 4.7. Body Length and Fluorescence Quantification of Age Pigment

Intestinal autofluorescence in *C. elegans* provided us a method to assay age pigment [30,40]. Worms treated with or without orange extract were prepared for measurement. After 5 days of the orange extract treatment, worms were firstly anaesthetized with NaN_3_ (20 μM) and then allocated to 1% agarose pads for visualization with a fluorescence microscope (CX23, Olympus, Tokyo, Japan) (excitation 360 nm, emission 430 nm). All pictures were taken at 100× magnification and Image J 8.5 software (NIH, Bethesda, MD, USA) was used to measure the relative fluorescence intensity and body length of the worms. All determinations were carried out in triplicate.

### 4.8. Stress Resistance Assay

#### 4.8.1. Thermotolerance Assay

The thermotolerance assay was carried out according to a previous report [41]. Similarly, *C. elegans* at the L4 stage were cultivated on plates with different concentrations of orange extract or plates without orange extract as the control group. *C. elegans* were kept on the treatments for 5 days followed by the exposure to thermal stress at 35 °C and dead worms were monitored every 2 h until all nematodes died. Each group included at least 90 animals. Experiments were repeated at least three times.

#### 4.8.2. Resistance to UV-B Radiation Assay

The worms (N ≥ 90) were treated with or without orange extracts for 5 days prior to induction of UV irradiation (120 mJ/cm^−2^) [42]. The number of surviving worms was counted daily by gentle prodding until all died. All assays were repeated in triplicate.

### 4.9. Intracellular ROS Levels

The levels of ROS in *C. elegans* were quantified via a specific fluorescent molecular probe (DCFH-DA) according to a previous report [29,43]. Prior to the experiment, nematodes were either treated with orange extract for 5 days or left untreated as the control group as described above. Approximately, 500 worms were collected and washed at least three times with M9 buffer (41 mM Na_2_HPO_4_, 15 mM KH_2_PO_4_, 8.6 mM NaCl, 19 mM NH_4_Cl). Subsequently, the worms were disrupted by ultrasonication and centrifuged at 3000× *g* for 3 min at 4 °C. After centrifugation, 50 µL of supernatant and 50 µL of 100 µΜ DCFH-DA dissolved in M9 buffer were transferred to a 96-well plate. Fluorescence was quantified at excitation/emission wavelengths of 485 nm/535 nm with a multi-mode microplate reader (Filter Max F5, Molecular Devices, Sunnyvale, CA, USA). Observations were recorded for 2 h at 10-min intervals. Determinations were performed in independent triplicate.

### 4.10. Determination of the Antioxidant Enzyme Activities and MDA Levels

Worms were treated with orange extract (0, 100, 200, and 400 mg/mL) for 5 days as described above. SOD and CAT activities as well as MDA contents were measured following the instructions of their corresponding kits. Total protein contents were determined by the bicinchoninic acid (BCA) assay kit. Results were normalized by protein contents represented as U/mg protein, U/mg protein, and nmol/mg protein, respectively. All of the assays were repeated at least three times.

### 4.11. Real-Time RT-PCR

Total RNA was extracted from approximately 1000 worms using Trizol reagent. The quality and quantity of RNA was assessed with Nucleic acid/Protein analyzer (DU730, Beckman Coulter, Inc., Fullerton, California, USA) and gel electrophoresis [44]. Complementary DNA (cDNA) was obtained using the PrimeScript™ RT Reagent Kit (Takara Biotechnology, Dalian, China) under the following conditions: 37 °C for 15 min, and then to 85 °C for 5 s. A Bio-Rad MiniOption™ Real Time PCR Detection System (Bio-Rad, Hercules, CA, USA) was used to analyze quantitative real-time PCR with SYBR Green Super-mix (Takara Biotechnology, Dalian, China). The expression levels of genes were analyzed using the 2^−ΔΔCt^ method. The primer sequences used for real-time PCR are listed in Table S1.

### 4.12. Statistical Analysis

All data were reported as mean ± standard deviation (SD) from at least three replicates. The graphs were depicted using the GraphPad INSTAT software (GraphPad software, San Diego, CA, USA). The statistical significance of the observed differences was evaluated using the IBM SPSS statistical software 21.0 (SPSS Inc., Chicago, IL, USA) with a *p*-value < 0.05. A Kaplan-Meier analysis and log-rank test were carried out to analyze the lifespan of *C. elegans*.

## 5. Conclusions

Supplementation with orange extract increased the mean lifespan of N2 wild-type *C. elegans* in a dose-dependent manner. Meanwhile, orange extracts improved the resistance against thermal and UV-B stresses, motility, and antioxidant-related enzyme activities (SOD and CAT) in the *C. elegans* model. Age pigment accumulation, contents of MDA, and intracellular ROS levels decreased after orange extract treatment. Furthermore, orange extract upregulated the mRNA expression of *sod-3*, *gst-4*, *daf-16*, *sek-1*, and *skn-1* and downregulated the mRNA expression of *age-1* in *C. elegans*. These results highlight that orange extract has a beneficial effect on *C. elegans*’ lifespan, making it a potential anti-aging candidate in the food industry.

## Figures and Tables

**Table 1:** Effect of orange extracts on thee lifespan of N2 wild-type *C. elegans* (mean ± SD, *n* = 3).

Group	Number	Mean Lifespan (Days)	% of Control	Maximum Lifespan (Days)
Control	113 ± 3	20.91 ± 1.60 ^a^	100.0 ± 7.6 ^a^	25.67 ± 2.08 ^a^
100 mg/mL	110 ± 5	23.10 ± 1.95 ^b^	110.5 ± 9.3 ^b^	28.00 ± 2.00 ^a,b^
200 mg/mL	106 ± 5	24.67 ± 2.26 ^b^	118.0 ± 10.8 ^b^	30.00 ± 2.65 ^a,b^
400 mg/mL	114 ± 3	26.38 ± 2.06 ^c^	126.2 ± 9.8 ^c^	32.33 ± 2.87 ^b^

Values with different letters in the same column indicated statistical significance (*p* < 0.05).

**Table 2:** Effect of orange extracts on heat shock of N2 wild-type *C. elegans* (mean ± SD, *n* = 3).

Group	Number	Mean Lifespan (h)	% of Control	Maximum Lifespan (h)
Control	94 ± 3	11.58 ± 1.02 ^a^	100.0 ± 9.3 ^a^	15.33 ± 1.15 ^a^
100 mg/mL	95 ± 2	12.71 ± 0.99 ^b^	109.8 ± 9.1 ^b^	19.33 ± 1.15 ^b^
200 mg/mL	97 ± 2	13.81 ± 1.24 ^b^	119.3 ± 11.4 ^b^	21.33 ± 1.15 ^b^
400 mg/mL	96 ± 6	15.68 ± 1.09 ^c^	135.5 ± 10.0 ^c^	21.33 ± 1.15 ^b^

Values with different letters in the same column indicate statistical significance (*p* < 0.05).

**Table 3:** Effect of orange extracts on UV-B irradiation of N2 wild-type *C. elegans* (mean ± SD, *n* =3).

Group	Number	Mean Lifespan (Days)	% of Control	Maximum Lifespan (Days)
Control	101 ± 5	4.53 ± 0.52 ^a^	100.0 ± 5.1 ^a^	6.67 ± 0.58 ^a^
100 mg/mL	92 ± 1	4.94 ± 0.71 ^a,b^	109.1 ± 9.7 ^a,b^	7.67 ± 0.58 ^a^
200 mg/mL	93 ± 1	5.52 ± 0.77 ^b,c^	122.0 ± 10.7 ^b,c^	9.00 ± 1.00 ^b^
400 mg/mL	93 ± 7	6.16 ± 0.90 ^b^	136.1 ± 13.6 ^b^	10.33 ± 0.58 ^c^

Values with different letters in the same column indicate statistical significance (*p* < 0.05).

## Supplementary Materials

The following are available online, Table S1: Primers used for quantitative PCR analysis.
