# Supplementary material for: Effects of Orange Extracts on Longevity, Healthspan, and Stress Resistance in Caenorhabditis elegans
Source: Molecules. 2020 Jan 15;25(2):351. doi: 10.3390/molecules25020351 (PMC7024185; doi:10.3390/molecules25020351)
Supplement: Supplementary file 1 [file molecules-25-00351-s001.pdf]

Table 1. Primers used for quantitative PCR analysis.

Gene	Gene ID	Forward Primer（5′-3′）	Reverse Primer（5′-3′）	Annealing Temperature (℃)	Fragment size (bp)	Primer Efficiency (%)
actin-1	179535	TCGGTATGGGACAGAAGGAC	CATCCCAGTTGGTGACGATA	59	108	94.64
sod-3	181748	GGCTAAGGATGGTGGAGAAC	ACAGGTGGCGATCTTCAAG	59	175	90.24
gst-4	177886	ATGCTCGTGCTCTTGCTGAG	GACTGACCGAATTGTTCTCCAT	59	163	89.75
daf-16	172981	TCAAGCCAATGCCACTACC	TGGAAGAGCCGATGAAGAAG	59	161	90.36
skn-1	177343	GACGTCAATTTATGGAGTGTCG	GAAGATGTTTTGTCGTGATCCG	59	137	92.56
sek-1	181043	ATGCTCGGTGAGTATTGG	TCATTGATAAACCGAGCC	59	165	93.96
age-1	174762	CCTGAACCGACTGCCAATC	GTGCTTGACGAGATATGTGTATTG	59	144	91.97

Highlights

- Orange extracts significantly promote longevity and stress resistance of *C. elegans*;
- Orange extracts promote the healthspan by improving motility, decreasing lipofuscin and intracellular ROS levels without damaging fertility in *C. elegans*;
- The antioxidant enzyme activities of SOD and CAT are enhanced, while the MDA contents are diminished in *C. elegans*;
- Insulin/ insulin-like growth factor signaling and mitogen-activated protein kinase;
- pathway pathways partly involve in the longevity mechanism of orange extracts.
